# Primary complement and antibody deficiencies in autoimmune rheumatologic diseases with juvenile onset: a prospective study at two centers

**DOI:** 10.1186/s12969-015-0050-8

**Published:** 2015-11-21

**Authors:** Mihaela Spârchez, Iulia Lupan, Dan Delean, Aurel Bizo, Laura Damian, Laura Muntean, Maria Magdalena Tămaș, Claudia Bolba, Bianca Simionescu, Cristina Slăvescu, Ioana Felea, Călin Lazăr, Zeno Spârchez, Simona Rednic

**Affiliations:** 2nd Department of Paediatrics, Iuliu Hatieganu University of Medicine and Pharmacy, 3-5 Crisan Street, Cluj-Napoca, 400177 Romania; Molecular Biology Center, Interdisciplinary Research Institute on Bio-Nano-Sciences, Babes-Bolyai University Cluj-Napoca, Cluj-Napoca, Romania; Emergency Children’s Hospital, Cluj-Napoca, Romania; Rheumatology Department, Emergency Clinical County Hospital, Cluj-Napoca, Romania; Department of Rheumatology, Iuliu Hatieganu University of Medicine and Pharmacy, Cluj-Napoca, Romania; 3rd Department of Internal Medicine, Iuliu Hatieganu University of Medicine and Pharmacy, Cluj-Napoca, Romania

**Keywords:** Primary complement, Antibody deficiency, Juvenile idiopathic arthritis, Juvenile onset systemic lupus erythematosus

## Abstract

**Background:**

Our aim was to investigate the prevalence and clinical relevance of inherited complement and antibody deficiency states in a large series of patients with various autoimmune rheumatologic diseases (ARD) with juvenile onset.

**Methods:**

A total number of 117 consecutive patients from 2 tertiary referral hospitals were included in the study. All patients underwent genetic screening for type I C2 deficiency and C4 allotyping. Serum levels of immunoglobulin classes measured systematically throughout their regular medical care were recorded retrospectively.

**Results:**

Our cohort of patients included 84 with juvenile idiopathic arthritis (JIA), 21 with systemic lupus erythematosus (SLE), 6 with systemic vasculitis, 2 with juvenile scleroderma, 2 with idiopathic uveitis, 1 with mixed connective tissue disease and 1 with SLE/scleroderma overlap syndrome. We have found 16 patients with evidence of primary immunodeficiency in our series (13.7 %), including 7 with C4 deficiency, 5 with selective IgA deficiency, 3 with C2 deficiency and 2 with unclassified hypogammaglobulinemia (one also presented C4D). Of the 84 patients with JIA, 4 (4.8 %) had a complement deficiency, which was less prevalent than in the SLE cohort (23.8 %), but all of them have exhibited an aggressive disease. Most of our patients with primary antibody deficiencies showed a more complicated and severe disease course and even the co-occurrence of two associated autoimmune diseases (SLE/scleroderma overlap syndrome and SLE/autoimmune hepatitis type 1 overlap).

**Conclusions:**

Our findings among others demonstrate that complement and immunoglobulin immunodeficiencies need careful consideration in patients with ARD, as they are common and might contribute to a more severe clinical course of the disease.

## Background

Primary immunodeficiencies (PIDs) are an inherited group of over 200 disorders that affect distinct components of the innate and adaptive immune system and predispose affected individuals to increased rate and severity of infection, immune dysregulation with autoimmune disease and malignancy [1, 2]. Although they are generally recognized as rare disorders, higher prevalence are expected with the improvement of PIDs analysis [3]. Unfortunately, PIDs are widely underdiagnosed and undertreated [3]. Domain experts have done international collaborative efforts recently in order to increase awareness about the importance of early diagnosis and access to optimal care in these patients [3].

A significant proportion of patients presenting with an autoimmune condition have an underlying PID disorder that may not be clinically relevant [4] yet it may contribute to a more aggressive and worse prognosis [5]. Conversely, at present, strong evidence exist in favor of the notion that PIDs are more prevalent in patients with autoimmune conditions than in general population. Selective immunoglobulin A deficiency (SIgAD), the most common PID, has an estimated incidence ranging between 1:143 and 1:875 in the general European population, and much lower among Asian populations [6]. On the other hand, Liblau et al. reported SIgAD in 4.3 % of patients with juvenile idiopathic arthritis (JIA) [7], whereas Cassidy et al. identified SIgAD in up to 5.2 % of children with systemic lupus erythematosus (SLE) [8]. Other antibody deficiency syndromes have also been reported in association with autoimmune rheumatologic disorders (ARDs) as well. Complement deficiencies involving components of the classical pathway (C1, C4 or C2) have been recognized as one of the strongest genetic risk factor for SLE [9, 10], but have also been described with an increased frequency in rheumatoid arthritis (RA) population [11]. However, studies that specifically address the prevalence of complement deficiencies in JIA patients, the most common childhood rheumatic condition, are limited. One study of 35 JIA patients has reported a prevalence of 14.3 % for C4 deficiencies [12] and in another two studies, C4 allotyping was considered in JIA population in order to find genetic susceptibility factors for the disease [13, 14].

The traditional diagnostics of complement deficiencies using serum C3 and C4 levels and CH50 activity was demonstrated not to be adequate to detect C4 and/or C2 deficiency in one study of Boeckler et al. [15] on SLE patients, allowing the diagnosis in a limited number of cases. Instead, the authors recommend C4 protein allotyping and genetic screening for type I C2 deficiency to be performed in investigative studies of primary complement deficiency states. According to Johnson et al. [16] this molecular bias is the most common cause of inherited C2 deficiency, occurring in over 90 % of C2- deficient individuals.

Therefore, we performed a prospective study in order to assess the prevalence and clinical relevance of inherited complement and antibody deficiency states in a large series of patients with various ARDs with juvenile onset.

## Methods

### Study population

This was a prospective observational cohort study. A total number of 117 consecutive patients with paediatric-onset autoimmune rheumatologic diseases (ARD) were included in the study. Our cohort of patients was recruited from 2 tertiary referral hospitals for rheumatologic patients across Cluj-Napoca, Romania, over 1 year (Emergency Children’s Hospital and the Rheumatology Unit of the Emergency Clinical County Hospital). The study was approved by the local Ethical Committee of the Iuliu Hatieganu University of Medicine and Pharmacy. Written informed consent has been obtained prior to their inclusion in the study as appropriate.

The onset of disease before 16 years of age and a well define diagnosis of ARD based on the revised ILAR criteria for JIA [17], new SLICC classification criteria for classification of SLE [18], EULAR/PRINTO/PRES classification criteria for childhood vasculitis [19], PRES/ACR/EULAR provisional classification criteria for juvenile systemic sclerosis (jSSc) [20] were the inclusion criteria. Patients with recent blood transfusions and incomplete or unavailable medical records were excluded from this study.

### Data collection

Medical records were obtained for evaluation from the attending physician. Demographic characteristics, family history of autoimmune diseases, PIDs and consanguinity, patient medical history (in particular infectious events, other associated autoimmune conditions, allergic diseases), diagnosis and history of present illness, drug therapy history since disease onset, associated conditions and complications were recorded for each patient. With regard to infections, we were interested in those that would arouse suspicion of PID: recurrent bacterial infections, mycobacterial infections, chronic mucocutaneous or invasive fungal infections and other opportunistic infections.

Furthermore, patient’s charts were retrospectively reviewed for the immunological investigations performed as part of the diagnosis process, before initiating immunosuppressive therapy and afterwards, during the period of follow-up. Therefore, we registered the following immunological parameters: total serum levels of IgG, IgA, IgM, IgE, C3, C4, circulating immune complexes (CIC) and rheumatoid factor (RF). All patients included in the study have had C3, C4 and Ig levels tested at the diagnosis of AID. When available, serum levels of IgG2 and IgG4 were also noted. In addition, the autoantibodies profile of patients focusing on the antinuclear antibodies (ANA) panel, anti-cyclic citrullinated peptide (CCP) antibodies, anti-dsDNA antibodies (dsDNA), antiphospholipid antibodies (APLAs) and organ-specific autoantibodies [anti-endomysium (EmA), anti-gastric parietal cells (GPC), anti-smooth muscle (ASMA), anti-mitochondrial (AMA), anti-liver-kidney microsomal (LKM1), anti-thyroid peroxidase (TPO)] was recorded too. The latter tests were performed depending on the clinical symptoms and anamnesis, according to the treating physician.

Reference ranges for the immunological tests analysed by immunoturbidimetry were as follows: 90–180 mg/dl for C3, 10–40 mg/dl for C4 and <18 IU/ml for RF. The serum levels of Ig were interpreted based on patient’s age. Autoantibodies test results were considered on their relation to the reference range of specific laboratory where the tests were done. The diagnosis of PID was established based on the new clinical diagnosis criteria for the European Society for Immunodeficiencies (ESID) registry [21]. Accordingly, the clinical criteria for a probable diagnosis of SIgAD were (1) at least one of the following: increased susceptibility to infection, autoimmune manifestations, affected family member; (2) diagnosis after 4th year of life; (3) undetectable serum IgA (when measured with nephelometry less than 0.07 g/L) but normal serum IgG and IgM (measured at least twice); (4) secondary causes of hypogammaglobulinaemia have been excluded; and (5) normal IgG antibody response to vaccination [21]. Unclassified hypogammaglobulinaemias was established in respect to the following criteria: (1) one of the following: recurrent infections, autoimmune phenomena (especially cytopenias), lymphoproliferation/ lymphoma; (2) marked decrease of at least one of IgG, IgG subclass (es), IgA or IgM levels (measured at least twice); (3) secondary causes of hypogammaglobulinaemia have been excluded; (4) normal isohaemagglutinins or/ and antibody response to vaccines; and (5) normal T-cells and normal naive T cells.

### Genetic analyses

Blood sample collection for the genetic analyses was performed during the routine visits in the hospital. Genomic DNA was extracted from peripheral blood using a Wizard® Genomic DNA Purification kit (Promega) according to manufacturer instructions with minor modifications.

#### Detection of type I C2 deficiency

PCR technique was used to detect the type I C2 deficiency (28 bp deletion that removes last 9 bp of exon 6 and first 19 bp of the intron 6, the translated protein is truncated). The PCR reactions were performed in 25 μl containing 1.5 mM MgCl2, 0.2 mM dNTPs, 0.5 μM of each primer, 1U of Dream Taq polymerase (ThermoScientific) and 100 ng of gDNA. The primers and PCR amplification program used were described previously [15]. The PCR products were detected by agarose gel electrophoresis (2 %).

#### Detection of C4 Deficiencies

The PCR reactions were performed in 25 μl as described above for C2 deficiency. The primers used for PCR amplification were described previously [22]. A touchdown PCR was performed starting with initial denaturation at 95 °C for 4 min, followed by 6 cycles of 94 °C for 30 s, 68 °C - 1 °C/cycle, 72 °C for 40 s; and 35 cycles of 94 °C for 30 s, 63 °C for 1 min, 72 °C for 40 s; final extension 5 min at 72 °C. The PCR products were detected by agarose gel electrophoresis (1.5 %). Patients with normal C4 alleles produced 2 fragments (377 and 578 bp) or no PCR amplification at patients with C4 deficiencies.

### Statistical methods

Numerical variables such as age and disease duration were summarized by mean, median and range. Dichotomous variables were provided as frequency (in percentage). The overall infectious complication rate express the percent of patients with significant infectious events in different groups. Small subsets of patients with PID did not allowed us to make any meaningful comparisons.

## Results

Our cohort included 84 patients with JIA (44 % female, 30 oligoarticular JIA, 23 polyarticular JIA, 18 systemic-onset JIA, 9 enthesitis-related arthritis, 1 psoriatic arthritis, 3 undifferentiated arthritis), 21 patients with SLE (90 % female), 6 patients with systemic vasculitis (83 % female), 2 patients with juvenile scleroderma (one boy with localised scleroderma and one girl with jSSc), 2 patients with idiopathic uveitis (both boys), 1 girl with mixed connective tissue disease (MCTD) and 1 girl with SLE/localized scleroderma overlap syndrome. All of them were Caucasions of European ancestry. Mean age at autoimmune disease onset was 8 years old with range between 0.7 and 16. Median duration of follow-up was 4 years, with range between 0.5 and 40 years.

We have found 16 patients with evidence of PID in our series. The distribution of PIDs among various juvenile-onset ARDs are presented in Table 1.

**Table 1:** Prevalence of PIDs among the 117 patients with various juvenile-onset autoimmune rheumatologic diseases

	Total (*n* = 117)	JIA (*n* = 84)	SLE (*n* = 21)	Vasculitis (*n* = 6)	MCTD (*n* = 1)	Scleroderma/SLE overlap syndrome
	n (%)	n (%)	n (%)	n (%)		(*n* = 1)
PIDs	16 (13.7)	6 (7.2)	6 (28.6)	2 (33.3)^a^	1	1
C4D	7 (6)	3 (3.6)	3 (14.3)	1 (16.6)	0	0
C2D	3 (2.6)	1 (1.2)	2 (9.5)	0	0	0
SIgAD	5 (4.3)	2 (2.4)	1 (4.8)	0	1	1
Unclassified hypogamma globulinaemia	2 (1.7)	0	0	2 (33.3)	0	0

*PID* primary immunodeficiency, *C4D* C4 complement deficiency, *C2D* C2 complement deficiency, *SIgAD* selective immunoglobulin A deficiency

^a^One patient with vasculitis has been diagnosed with hypogammaglobulinemia, also complete C4B deficiency

Out of the 16 PID patients, only 2 were boys. With the exception of patient 6 and 7, our PIDs patients had no family history of PID. The overall infectious complication rate was 43.75 % in the PID patients and only 5 % in the cohort with no evidence of PID. The age at autoimmune disease onset in the PID patients has ranged between 0.7 and 16 years of age. One SIgAD patient had an infantile onset and four patients had the onset of symptoms at 2–7 years of age. Duration of follow-up in the PID group was between 0.5 and 30 years. The diagnosis of inherited antibody deficiency was established concomitant with the AID diagnosis in patient 1, 3, 4, 5 and 6 or previous to AID onset in patient 2 (at 8 years old) and patient 7 (at 4 years old). The primary complement deficiencies were diagnosed at the time of this study in all patients. Detailed clinical and immunological characteristics of our PID patients and also the significant infection episodes are described in Table 2.

**Table 2:** Clinical and immunological characteristics of the 16 patients with PID in our series

		PID	Autoimmune disease with clinical and laboratory features	Age at AID onset	Duration of follow up (years)	Autoantibody profile	Infectious events indicative of PID
			Associated conditions and complications				
1	F	SIgAD	SLE/scleroderma overlap syndrome	5 for LS 16 for SLE	24	ANAs	Urinary tract infections
			Raynaud phenomena, generalized morphea, lupus nephritis, microangiopathic haemolytic anaemia, secondary antiphospholipidic syndrome, systemic arterial hypertension, diffuse interstitial pulmonary fibrosis, pulmonary hypertension			dsDNA	Pneumonias from an early age
						Sm, RNP, Histone	
						Nucleosome	
2	F	SIgAD	SLE associated with autoimmune hepatitis type 1	16	2	ANAs, Sm, U1 RNP	Urinary tract infections prior to AID diagnosis
			lupus malar rash, photosensitivity, synovitis, lymphopenia, low complement			Ribosomal P protein	
						Nucleosome, ACA, ASMA	
3	F	SIgAD	Systemic-onset JIA: polyarticular course, chronic bilateral iridocyclitis with bilateral cataract formation and band keratopathy, loss of vision	0.7	16	ANAs^a^	No
4	F	SIgAD	MCTD: polyarthritis, livedo reticularis, Raynau d’s phenomenon, alopecia, Gottron’s papules, rash of dermatomyositis, one psychotic episo de, aseptic necrosis of the hip, direct Coombs ’s test, intermittent low C3 Allergic Asthma	14	13	ANAs,	Esophageal candidiasis after AID therapy
						dsDNA	
						U1 RNP	Urinary tract infections prior to AID diagnosis
						ANCAs	
5	M	SIgAD	Undifferentiated JIA: oligoarthritis, positive RF Allergic Asthma	9	1.5	No	No
6	F	Unclassified hypogammaglobulinaemia Complete C4BD	Systemic vasculitis (ANCA negative): fever, myalgia, a rthralgia, livedo reticularis, maculopapular rash, III and VI cranial nerve palsy, transient ischemic attack, haemorrhagic stroke with right hemiparesis, thrombocytopenia, nodular regenerative hyperplasia of the liver, splenomegaly, growth retardation	2.5	4	No	Pneumonias prior to AID diagnosis
7	F	Unclassified hypogammaglobulinaemia	Systemic vasculitis (ANCA negative): fever, myalgia, livedo reticularis, cutaneous nodules, systemic arterial hypertension, abdominal recurrent pain, massive intestinal bleeding; bicuspid aortic valve disease with aortic valve insufficiency	6.5	2	No	Severe Epstein-Barr virus-induced acute infectious mononucleosis at the age of 3
8	F	Homozygous C2D	SLE: photosensitive lupus rash, oral ulcers, synovitis, perniosis, serum high CIC levels	12	2.5	ANAs	No
						SS-A, Ro 52	
9	F	Heterozygous C2 D	SLE: malar rash, synovitis, lupus nephritis, thrombocytopenia, leukopenia, hemolytic anemia, low serum C3 and C4 levels, serum high CIC levels, renal transplantation	12	2.5	ANAs^b^	No
						dsDNA	
10	M	Heterozygous C2 D	Seronegative polyarticular JIA, Bronchogenic cyst with surgical resection in infancy	8	11	ANAs in low titers^b^	No
11	F	Complete C4B deficiency	SLE: lupus nephritis, polisynovitis, photosensitive lupus rash, low serum C3 and C4 levels, serum high CIC levels, secondary antiphospholipidic syndrome	12	1.5	ANAs, dsDNA, Histone, pANCA	No
						ACA, β2 GP	
12	F	Complete C4B deficiency	Systemic-onset JIA: fever, polyarthritis, evanescent rash, myalgias	7	9	No	No
13	F	Complete C4B deficiency	Oligoarticular JIA	1.5	0.5	No	No
14	F	Partial C4A deficiency	SLE: Raynaud phenomena, lupus nephritis, systemic arterial hypertension, secondary vasculitis, synovitis, livedo reticularis, pericarditis, secondary antiphospholipidic syndrome, low serum C3 and C4 levels	10	30	ANAs, Ro (SS-A)	Pulmonary tuberculosis at 35 years old
						dsDNA, pANCA	
						ACA- IgG	
15	F	Partial C4A deficiency	SLE associated with autoimmune hepatitis type 1: fever, synovitis, lupus malar rash, oral ulcers, serositis, haemolytic anaemia, secondary cutaneous vasculitis, Libman-Sacks endocarditis	15	5	ASMA, ACA, ANAs^§^	Pulmonary tuberculosis at 15 years old
16	F	Partial C4A deficiency	Undifferentiated JIA: oligoarthritis, positive RF, bilateral chronic iridocyclitis with loss of vision	7.3	3	ANAs^§^	No

*AIH* autoimmune hepatitis, *ACA* anti-cardiolipin antibodies, *ANAs* antinuclear antibodies, *C4D* C4 deficiency, *C2D* C2 deficiency; *CVID* common variable immunodeficiency, *dsDNA* antibodies to double stranded DNA, *F* female, *Ig* Immunoglobulin, *JIA* Juvenile Idiopathic Arthritis, *LS* localized scleroderma, *M* male, *MCTD* mixed connective tissue disease, *RF* rheumatoid factor, *SIgAD* selective IgA deficiency, *SLE* systemic lupus erythematosus, *β2 GP* anti-β2-glycoprotein I antibodies

^a^Negative ANA profile test (immunoblot), despite high titers of ANAs on indirect immunofluorescence (1/1280; homogeneous pattern)

^b^ANAs profile not available

Unclassified hypogammaglobulinaemia has been designated in two siblings (patient 6 and 7), both with ANCA- negative systemic vasculitis. Both sisters have demonstrated persistently reduced IgM [the mean IgM level during the disease course was 11 mg/dl (range 5–22) in patient 6 and 27 mg/dl (range 20–35) in patient 7; the normal range for their age was 23–190 mg/dl in patient 6 and 50–260 mg/dl in patient 7] and IgA [the mean IgA level was 9 mg/dl (range 2–18) in patient 6 and 20 mg/dl (18–43) in patient 7; the normal range for their age was 23–190 mg/dl], decreased IgG4 (<0.6 mg/dl in patient 6 and 2 mg/dl in patient 7; normal range 3–157 mg/dl). Patient 6 also had reduced IgG2 (61 mg/dl, normal range 65–220 mg/dl) and complete C4BD. Total serum IgG levels were not constantly reduced below 500 mg/dl in patients 6 and 7, although in patient 6 they were occasionally decreased (the lowest value was 326 mg/dl).

Homozygous C2D was identified in one girl (patient 8) with SLE onset at 12 years of age, prominent photodermatitis and articular involvement and positive anti-Ro antibodies. Two other patients had heterozygous C2D (patient 9 and 10). Patient 9 was a girl with SLE and significant nephritis within 2 years after onset who underwent renal transplantation. Patient 10 was a boy with RF negative polyarticular JIA, with positive ANA in low titres, with still on-going active arthritis eleven years after onset.

Complete C4B deficiency was found in four patients. Apart from patient 6, there was one SLE female patient (patient 11), one systemic JIA patient (patient 12) and one girl with oligoarticular JIA (patient 13). Of the 3 patients with partial C4A deficiency, one had an undifferentiated form of JIA (patient 16) and two patients had SLE (patient 14 and 15). Notably, patient 15 had associated SLE and autoimmune hepatitis type I.

Patients with SIgAD had the following diagnoses: one SLE/scleroderma overlap syndrome (IgA between 0 and 5 mg/dl), one SLE associated with autoimmune hepatitis type I (IgA between 0 and 7 mg/dl), one systemic-onset JIA (IgA between 2 and 6 mg/dl), one MCTD (IgA = 5 mg/dl), one oligoarticular JIA (IgA = 4 mg/dl). Of note, two of them associated allergic asthma.

Notably, the association of overall PID was greater in the SLE patient population relative to the JIA group of patients (28.6 % versus 7.2 %). In particular, C4D, C2D and also SIgAD were more prevalent in SLE group of patients than in the JIA cohort.

Among the 6 patients with positive RF in our JIA cohort, 2 were diagnosed with PID: patient 5 with SIgAD and patient 16 with partial C4AD. Acute anterior uveitis occurred in 9 JIA patients (2 with PID and 7 without PID). Infectious events, associated autoimmune conditions other than JIA, complications including macrophage activation syndrome and secondary amyloidosis occurred in very low numbers only in JIA population withough PID. The associated autoimmune diseases found in our JIA patients were type I diabetes mellitus and autoimmune thyroiditis.

Two SLE-PID patients presented with pulmonary tuberculosis at 14 years (patient 15) and 35 years of age (patient 14), respectively. It is important to note that in both cases there were no other risk factors for acquiring the infection. Moreover, two patients with SLE and PID associated autoimmune hepatitis type I (patient 2 with SIgAD and patient 15 with partial C4A D).

The small number of patients with PID did not allowed us to make any meaningful comparisons regarding clinical and immunological features of the disease.

## Discussion

Over the past decades, there has been an increasing interest in the field of immunopathology providing a better understanding of the mechanisms that underlie diseases such as immunodeficiency, autoimmunity, autoinflammation or cancer. This was reflected in adding new disease entities, modern diagnostic strategies and novel immunotherapies that are being constantly developed. Although the spectrum of PIDs has been steadily expanding, a substantial number of patients remain undiagnosed.

We believe that this is the largest JIA cohort of patients investigated for both primary C2 and C4 complement components deficiencies. Of the 84 patients with JIA evaluated in the present study, 4.8 % have had genetic evidence of early complement deficiency, which was less prevalent than in our SLE patients (23.8 %). This may be regarded as a significant proportion, in comparison with the prevalence of complement deficiencies in general population, which has been calculated to be about 0.03 % (excluding MBL deficiency) [23]. In our series, C4D has been associated with JIA in 3.6 % of the cases, unlike the higher prevalence of 14.3 % reported in one previous study [12], using the same previously described primers for PCR amplification [22]. This variability may be explained by the difference in the total number of patients investigated (84 vs 35) or different genetic background. The older studies offer divergent results regarding the association between JIA and null alleles at the C4A and C4B locus [13, 14]. Heterozygous C2D was found in only one patient with seronegative polyarticular JIA. There is a paucity of studies relating to complement deficiencies in JIA. Gilliam et al. believe that many researchers did not considered this association, because polyarticular and oligoarticular JIA does not have systemic vasculitis [12]. But, later on, the same authors demonstrated complement activation with a dominant role of the classical pathway in both polyarticular and oligoarticular subtypes of JIA [24]. In addition, another recent study found classical pathway up-regulated in systemic JIA too [25]. This data suggest greater than anticipated contributions of the complement pathways in the pathogenesis of JIA, regardless of the subtype. Given all that, it seems likely that dysregulation of the complement system in cases with defects of the pathway’s components may exacerbate the disease process, due to inappropriate activation of the system and impaired dissolution of immune complexes that cumulate and maintain the synovial inflammation. Indeed, all our complement deficient JIA patients have had an aggressive disease by chronic continuous or polyphasic arthritis (patient 10 and 12), severe bilateral chronic iridocyclitis with loss of vision (patient 16) and very early onset of the disease (1.5 years of age in patient 13). Remarkably, none of these patients had any clinical warning signs for PID, besides the occurrence of autoimmune manifestations.

We have also found complement deficiencies in the 23.8 % of the SLE patients and in another systemic vasculitis patient with concomitant unclassified hypogammaglobulinaemia and complete C4B deficiency. This was not surprising, since associations between systemic autoimmune diseases and any of the early components of the classical pathways are all recognised [23, 26]. For instance, homozygous C2 deficiency, which is the most frequent hereditary deficiency in classical complement pathway, was estimated to be associated with systemic autoimmune disease in around 15 to 40 % of the cases [27]. Deficiencies of C4 are very rare but associated with a higher prevalence of SLE (75 %) [26], and almost exclusively with the deficiency of C4A isotype [28]. In our SLE cases with C4D, 2 had a partial deficiency of the C4A isotype and one had a complete deficiency of C4B isotype. We did not observed in these patients a younger age at disease onset as previously noted [29]. Moreover, it has been suggested in complement deficient SLE patients less renal, pulmonary or pericardial involvement and more prominent annular photosensitive skin rashes [29]. On the contrary, we have noted severe lupus nephritis within two years after onset in two girls with heterozygous C2D and complete C4BD and mild lupus nephritis with a later onset during the disease course in the patient with partial C4AD. But patient 8 has adhered more closely to the earlier described pattern of SLE patients with homozygous C2D: dominate photosensitivity and articular involvement, no renal disease, presence of anti-Ro antibodies [26].

Primary antibody deficiencies that we have found in our cohort of patients consisted of 5 SIgAD cases and 2 unclassified hypogammaglobulinaemias in two siblings. Our data regarding the prevalence of SIgAD in our JIA and SLE patients were more consistent with the previous studies [5, 7, 8]. It is important to note that most of antibody deficient patients showed a more complicated and severe disease course, which may be, at least in part, an effect of the associated PID. Remarkably, one girl with localized scleroderma at the age of 5 years old has consecutively developed SLE with lupus nephritis, secondary antiphospholipidic syndrome, microangiopathic haemolytic anaemia, diffuse interstitial pulmonary fibrosis and pulmonary hypertension. Another SIgAD patient with a polyarthritis onset at the age of 14 developed over several years features of other connective tissue diseases, fulfilling the criteria of MCTD. Worthy of attention also are the sisters with systemic vasculitis from an early age with associated unclassified hypogammaglobulinaemias before immunosuppressive treatment was initiated, of which one had two stroke events at the age of 4 and recurrent cranial nerve palsies and the other had a severe episode of gastrointestinal haemorrhage. They could not be considered common variable immunodeficiency disorders (CVID) because total serum IgG levels were not constantly reduced below 500 mg/dl, even though patient 6 developed a variety of autoimmune manifestations characteristic of CVID according to recent relevant literature [30], including persistent autoimmune thrombocytopenia, livedo reticularis with negative APLAs, splenomegaly, nodular regenerative hyperplasia of the liver, skin lesions with histopathological pattern of interstitial granulomatous dermatitis in association with vasculopathy. The recently described genetic disorder “deficiency of ADA2” is being considered in both sisters, but yet unproven [31].

Unlike patients with SLE, systemic vasculitis and both patients with MCTD and SLE/scleroderma overlap syndrome, recurrent infectious events were not associated with PIDs in our JIA patients. We would like to stress that the available observation period for some of the JIA patients with PID (patient 5, 13 and 16) was relatively short. But, as it has been suggested before, the autoimmunity may be the only manifestation of a PID and that the absence of repeated infections is not sufficient to exclude a PID [4]. Conversely, another matter of concern is the confounding impact of longer duration of follow up after diagnosis in developing infections. That by itself may have been increased the risk of infections in some patients (patient 1 and 4) due to longer immunosuppressive therapies and hospitalizations.

A better understanding of this paradoxical relationship between autoimmunity and immunodeficiency is needed in order to provide more explanations about the contributions of PID to disease expressivity. Until than, the most relevant aspect of this relationship for the clinician remains the implications of PID identifying in the follow-up of these patients, which can be quite diverse and individualistic. Patients with a known complement deficiency should be carefully monitored for development of other potential complications or associated conditions involving complement activation. For instance, in a large cohort of C2-deficient SLE patients with long-term follow-up, a high frequency of severe cardiovascular damage was found that could not be explained by common risk factors [32]. Moreover, clinical situations of PID with overlap between increased susceptibility to infection and autoimmunity may require special considerations in selecting the immunosuppressive and biologic therapy, patient education and clinical monitoring. Finally, the immunoglobulin therapy, the mainstay of treatment for primary hypogammaglobulinemias with decreased IgG production, has also proved to be efficacious in specific autoimmune and inflammatory diseases [33].

To our knowledge, the co-existing of autoimmune hepatitis type 1, SLE and PIDs (SIgAD or C4AD) that we have encountered in patient 2 and 15, has not been reported in the literature so far. There have been few reported cases of either AIH associated with SLE [34–37] or AIH and SIgAD [38] or AIH and C4 deficiency [39] or AIH, SIgAD and primary Sjogren’s syndrome [40]. Between the two types of AIH, type 1 seems to be more often associated with other autoimmune diseases, while type 2 presents more commonly with IgA deficiency [41]. We acknowledge that AIH type 1 diagnosis in our SLE patients was made only on clinical, biochemical and serologic data and that the liver biopsy for a definitive diagnosis of AIH has not been done for objective reasons.

## Conclusion

In conclusion, a significant number of our JIA patients had genetic evidence for C2D or C4D and all of them have exhibited an aggressive disease. However, further studies are needed to decipher the implications of this association on disease expression and outcome of these patients. Moreover, most of our patients with primary antibody deficiencies including SIgAD and unclassified hypogammaglobulinaemias showed a more complicated and severe disease course and even the co-occurrence of two autoimmune diseases (SLE/scleroderma overlap syndrome and SLE/AIH type 1 overlap). We also report the first two cases of SLE/AIH type 1 overlap associated with SIgAD and C4AD, respectively. Our findings among others demonstrate that complement and immunoglobulin immunodeficiencies need careful consideration in patients with ARDs, as they are common and might contribute to a more severe clinical course of the disease.

## Abbreviations

ACR: American College of Rheumatology
ARD: Autoimmune rheumatologic disorder
C2D: C2 deficiency
C4D: C4 deficiency
ESID: European Society for Immunodeficiencies
EULAR: European League against Rheumatism
ILAR: International League of Associations for Rheumatology
JIA: Juvenile idiopathic arthritis
PID: Primary immunodeficiency
PRES: Paediatric Rheumatology European Society
PRINTO: Paediatric Rheumatology International Trials Organisation
RA: Rheumatoid arthritis
SIgAD: Selective immunoglobulin A deficiency
SLE: Systemic lupus erythematosus
SLICC: Systemic Lupus International Collaborating Clinics
